# Promising Therapeutic Strategies for Mesenchymal Stem Cell-Based Cardiovascular Regeneration: From Cell Priming to Tissue Engineering

**DOI:** 10.1155/2017/3945403

**Published:** 2017-02-20

**Authors:** Seung Taek Ji, Hyunyun Kim, Jisoo Yun, Joo Seop Chung, Sang-Mo Kwon

**Affiliations:** ^1^Laboratory for Vascular Medicine and Stem Cell Biology, Convergence Stem Cell Research Center, Medical Research Institute, Pusan National University School of Medicine, Yangsan, Republic of Korea; ^2^Department of Internal Medicine, Medical Research Institute, Pusan National University School of Medicine, Busan, Republic of Korea

## Abstract

The primary cause of death among chronic diseases worldwide is ischemic cardiovascular diseases, such as stroke and myocardial infarction. Recent evidence indicates that adult stem cell therapies involving cardiovascular regeneration represent promising strategies to treat cardiovascular diseases. Owing to their immunomodulatory properties and vascular repair capabilities, mesenchymal stem cells (MSCs) are strong candidate therapeutic stem cells for use in cardiovascular regeneration. However, major limitations must be overcome, including their very low survival rate in ischemic lesion. Various attempts have been made to improve the poor survival and longevity of engrafted MSCs. In order to develop novel therapeutic strategies, it is necessary to first identify stem cell modulators for intracellular signal triggering or niche activation. One promising therapeutic strategy is the priming of therapeutic MSCs with stem cell modulators before transplantation. Another is a tissue engineering-based therapeutic strategy involving a cell scaffold, a cell-protein-scaffold architecture made of biomaterials such as ECM or hydrogel, and cell patch- and 3D printing-based tissue engineering. This review focuses on the current clinical applications of MSCs for treating cardiovascular diseases and highlights several therapeutic strategies for promoting the therapeutic efficacy of MSCs in vitro or in vivo from cell priming to tissue engineering strategies, for use in cardiovascular regeneration.

## 1. Introduction

The World Health Organization (WHO) announced that the leading cause of death among chronic diseases worldwide is ischemic cardiovascular diseases, such as stroke and myocardial infarction (MI) [1]. Cardiovascular disease is caused by fat accumulation, platelet aggregation, and blood clots formation in the lining of blood vessels. Cardiovascular disease encompasses a very broad range of conditions, such as heart diseases, including MI, hypertension, heart failure, arrhythmias, cardiomyopathy, and ischemic heart disease due to atherosclerosis progression and vascular disease including peripheral vascular disease and stroke [2].

The treatment approach for the majority of cardiovascular disease is to administer drugs, and some cases may require surgery such as coronary angioplasty with stent insertion into the narrowed blood vessel to normalize blood flow through the coronary artery and coronary artery bypass [3]. In addition, gene therapy has been applied to treat cardiovascular disease [4]. In particular, phase II clinical trials of therapeutic angiogenesis using gene therapy are in progress, and the method is expected to be available soon for clinical use. The incidence of cardiovascular disease has continued to increase, and aside from transplantation, other therapies, despite recent advances in heart treatments, cannot fundamentally remedy the major etiology of cardiovascular disease; thus, there is a limit to how much treatment outcomes can be improved with the current approaches [5]. Although various studies have been conducted to overcome the limitations of cardiovascular therapies, stem cell therapy using several types of stem cells such as hematopoietic stem cells (HSCs), mesenchymal stem cells (MSCs), cardiac stem cells (CSCs), and endothelial progenitor cells (EPCs) provides an alternative approach, and remarkable advances have been made in clinical and basic research [6].

Several reasons favor the clinical and therapeutic application of adult stem cells over embryonic stem cells (ESCs), including controlled proliferation, exceptional reliability, and a site-specific differentiation ability [7]. Among adult stem cells, MSCs are frequently used to treat the most common cardiovascular diseases. MSCs can be found in the bone marrow (BM), adipose tissue, umbilical cord blood (UCB), and many other tissues. They have self-renewing properties and are multipotent progenitor cells that can differentiate into various lineages such as osteocytes, chondrocytes, adipocytes, and myocytes [8–11]. MSCs also have immunomodulatory properties [12, 13]. In addition, MSCs are unlikely to lead to immune rejection because of their low expression of CD40, CD80, and CD86, as well as MHC class I molecules [14, 15]. The therapeutic benefit of this approach is based on the potency of secretion of beneficial cytokines and growth factors for tissue repair/regeneration, as well as the immunomodulation effect and/or their differentiation for regenerating damaged organs [16].

MSCs can be applied for cardiovascular regeneration and provide therapeutic benefit for cardiovascular disease. However, MSCs have several disadvantages regarding their therapeutic application, including their very low survival rate in vivo and integration rate into the host cells after transplantation [17]. Another limitation is the low accuracy in delivering the stem cells to the damaged site [18]. Various attempts have been made to improve the poor survival and longevity of engrafted MSCs. The first step in developing therapeutic strategies is the identification of more effective reagents for promoting the ability of stem cells via understanding stem cell niche modulators. An emerging promising therapeutic strategy is the preconditioning of MSCs before transplantation using cytokines and natural compounds that induce intracellular signaling or niche stimulation through paracrine mechanisms [19]. Another is a tissue engineering-based therapeutic strategy involving a cell scaffold, a cell-protein-scaffold architecture made of biomaterials such as ECM or hydrogel, and cell patch- and 3D printing-based tissue engineering, to enhance cell survival via cell-cell communication or cell-scaffold interactions [20].

This review focuses on promising cell therapeutic strategies for MSC therapy against cardiovascular diseases and introduces a variety of studies designed to promote the therapeutic efficacy of MSCs in vitro or in vivo from cell priming strategies to tissue engineering strategies for cardiovascular regeneration.

## 2. Characterization of MSCs

The term “mesenchymal stem cells” was introduced by Caplan in the early 1990s [21], although nonhematopoietic, “mesenchymal” precursor cells were first described in the 1970s by Friedenstein et al. as a population of bone marrow stromal cells capable of mesodermal differentiation and trophic support of hematopoiesis [22, 23].

MSCs are present in almost all tissues of the body and are located in the perivascular space [24]. They can also be isolated from other sources, such as adipose tissue [25–30], cartilage [31, 32], umbilical cord blood [33–36], and peripheral blood [37]. In addition, MSCs can be derived from many different organs and tissues, including the brain, spleen, liver, kidney, lung, bone marrow, muscle, thymus, and pancreas [38].

The Mesenchymal and Tissue Stem Cell Committee of the International Society for Cellular Therapy (ISCT) set minimal criteria in 2001 for defining MSCs. They must be plastic-adherent when maintained in standard culture conditions. Second, MSCs must express CD105, CD73, and CD90 and lack the expression of CD45, CD34, CD14, or CD11b; CD79*α* or CD19; and HLA-DR surface molecules as assessed by fluorescence-activated cell sorter analysis. Third, MSCs must be able to differentiate into osteoblasts, adipocytes and chondroblasts in vitro [39, 40]. Other surface markers generally expressed by MSCs include CD9, CD105 (SH2), CD73 (SH3/4), CD44, CD90 (Thy-1), CD71, CD106 (vascular cell adhesion molecule- [VCAM-] 1), CD166 (activated leukocyte cell adhesion molecule [ALCAM]), intercellular adhesion molecule- (ICAM-) 1, and CD29 [9, 14, 41, 42]. Stem cells are characterized by their ability to self-renew, clone, and differentiate into multiple tissues [6]. MSCs are a pluripotent cell type that can differentiate into several distinct lineages, including mesodermal lineage cells (osteoblasts, chondrocytes, and adipocytes) [9, 43, 44] and myogenic lineage [45]. Adipogenic differentiation is induced with FBS in medium supplemented with dexamethasone, insulin, isobutyl methyl xanthine, and indomethacin. The differentiation can be confirmed by the staining of lipid vacuoles with oil red O [46, 47] and measuring the levels of several proteins, including PPAR *γ*, fatty acid-binding protein aP2, and lipoprotein lipase [27]. For osteogenic differentiation, MSCs are treated with ascorbic acid, beta-glycerophosphate, and dexamethasone. The differentiation is confirmed increase in alkaline phosphatase activity and calcium deposition [47, 48]. Chondrogenic differentiation is induced by culture with serum-free medium supplemented with transforming growth factor-beta (TGF-*β*), resulting in an increase in the levels of highly glycosaminoglycan and type II collagen [46, 49].

Recently, in vivo studies demonstrated that human MSCs transdifferentiate into endoderm-derived cells and cardiomyocytes [50–52], and MSCs transdifferentiated into a cardiomyocyte-phenotype [53]. Animal preclinical studies of MSC administration in post-MI hearts revealed the ability of MSCs to engraft, differentiate, and produce substantial functional recovery [54–57]. In recent years, MSCs therapy has been translated to clinical trials for ischemic heart disease [58–60].

## 3. Clinical Trials Using MSCs against Cardiovascular Diseases

Cell-based treatments represent a new generation in the evolution of biological therapeutics. A prototypic cell-based therapy for heart failure using MSCs has successfully reached the pivotal phase III trials, indicating adequate safety and efficacy data from phases I and II trials. Successful phase III trials can lead to the approval of a new biologic therapy for regenerative medicine [61]. There have been about 43 clinical trials using MSCs in relation to cardiovascular regeneration registered with clinicaltrials.gov (Table 1, a web-based service by the US National Institute of Health). Among the MSC-based clinical trials, studies designed to treat cardiovascular disease represent a substantial proportion (14.8%) [62]. On the basis of the rigorous preclinical testing highlighted above that demonstrated the safety of MSC delivery to patients with cardiac disease, clinical trials have been initiated for both acute MI and ischemic cardiomyopathy. The results of a clinical trial of autologous MSC transplantation might aid in improving the long-term survival of patients with severe heart failure and significantly reduce hospitalizations for worsening angina [63]. A much smaller study revealed that both autologous BM MNCs and expanded BM MSCs reduced myocardial scarring by 3 months, indicating the stimulation of beneficial tissue remodeling [64]. The intracoronary administration of MSCs has been shown to have a minor benefit on the left ventricular ejection fraction [65], and a meta-analysis of cell therapies involving intracoronary administration found that there was no clinical benefit for left ventricular function [66]. The four clinical trials of plasmonic photothermal therapy (PPTT) using silica-gold nanoparticles demonstrated the significant regression of coronary atherosclerosis [67]. In an early stage study of patients with ICM, the transendocardial injections of allogeneic and autologous MSCs without a placebo control were both associated with low rates of treatment-emergent SAEs, including immunologic reactions [68]. Transendocardial stem cell injection with MSCs or BMCs appeared to be safe for patients with chronic ischemic cardiomyopathy and LV dysfunction [69]. The intramyocardial injection of autologous MSCs into akinetic but nonrevascularized segments produced comprehensive regional functional restitution, which in turn drove improvement in global LV function. These findings, although inconclusive because of the lack of a placebo group, have important therapeutic and mechanistic hypothesis-generating implications [70].

## 4. Understanding MSC Biology for Tissue Regeneration

### 4.1. Enhancing MSC Survival for Cardiovascular Regeneration

In ischemic sites of ischemia-reperfusion injuries, cardiomyocytes undergo apoptosis [71]. Accumulating evidence clearly indicates that activated Akt signals protect cardiomyocytes from apoptosis. Similarly, transduced Akt in MSCs significantly enhanced their stem cell function in an in vivo rat model [72]. Lim et al. investigated the pivotal role of Akt-transduced MSCs in the ischemic porcine heart [55]. When applied to MI, Akt-MSCs administration increased the left ventricular ejection fraction and decreased the area of MI. Notably, Akt-MSCs have an enhanced cell survival ratio with augmented expression levels of ERK and VEGF, suggesting that administering Akt-transduced MSCs might be a promising therapeutic strategy for treating human patients with cardiovascular diseases.

Epidermal growth factor (EGF) is a well-known cytokine involved in cell growth and vascular tissue repair. In general, EGF binds to the EGF receptor (EGFR) and activates extracellular-regulated kinase (ERK) and Akt-mediated intracellular signaling pathways, resulting in increased cellular activities, including cell adhesion, migration, proliferation, and cell survival. Recently, it was reported that MSCs stimulated by soluble EGF induced EGFR signaling and promoted the upregulation of migration and proliferation [73]. Fan et al. also studied the pivotal role of surface-tethered EGF in MSC survival [74]. The proportion of total ERK that was phosphorylated was strongly linked to improved cell spreading and cell survival in tEGF-polymer conditions. Under these severe culture conditions, tEGF-polymer protected MSCs from FasL and eventually increased the survival rate, suggesting that tethered EGF might have protective functions in transplanted MSCs during acute inflammatory reactions.

Vascular endothelial growth factor (VEGF) has a pivotal role in cardiovascular regeneration. When MSCs isolated from B6 mice were cocultured with VEGF peptide, the primed MSCs exhibited reduced levels of cellular stress and higher expression of prosurvival factors via phosphorylation of Akt and Bcl-xL. The administration of MSCs primed with VEGF peptide in an MI disease model resulted in improved cardiac function via enhanced cell engraftment and cell survival capabilities, indicating that VEGF protects MSCs from cellular stress, leading to enhanced cardiac function and cardiovascular regeneration.

### 4.2. Enhancing MSC Proliferation for Cardiovascular Regeneration

In the human body, the subpopulations of MSCs are very small. Healthy MSCs dramatically activate their self-renewal signaling pathway when required. After the onset of an injury caused by ischemic cardiovascular diseases, an insufficient MSC number leads to impaired tissue regeneration. Furthermore, the clinical use of MSCs for tissue regeneration has been limited mainly because they have a low proliferation rate and progressively lose their stem cell properties during in vitro expansion. To overcome these limitations, many research groups have studied the signaling cascades associated with MSC proliferation and identified pivotal modulators for MSC proliferation for use in the treatment of cardiovascular diseases [75].

Octamer-binding transcription factor 4 (Oct4) and sex determining region Y-box 2 (Sox2) are pluripotent stem cell-specific factors. The pivotal roles of these two factors in maintaining MSC stemness and proliferation have been studied [76, 77]. Although early passage MSCs express Oct4 and Sox2 at low levels, their expression levels decrease as the passage number increases. To improve MSC proliferation and stemness, human adipose tissue MSCs (ATMSCs) were transfected with Oct4 and Sox2 [78], and the MSCs that expressed Oct4 and Sox2 exhibited enhanced proliferative activity. This result was mediated by the upregulation of cyclin D1, indicating that the transition of cells from G1 to S phase might be accelerated.

The Wnt signaling pathway regulates cell proliferation and cell fate determination [79]. The canonical Wnt signaling pathway is a well-known Wnt signaling pathway involved in stem cell biology. Wnt ligands secreted from a cell can affect the cell itself (autocrine) or neighboring cells (paracrine), and they bind to their receptor, low-density lipoprotein receptor-related protein (LRP) 5/6. As a result, stabilized *β*-catenin is translocated to the nucleus, where it upregulates its downstream genes. Activation of the canonical Wnt signaling pathway in stem cells results in increased proliferation or self-renewal activity in ESCs, neural stem cells (NSCs) [80], and HSCs [81]. In addition to previous studies on stem cells, MSC regulation by Wnt signaling pathways has been investigated. When adult MSCs are treated with Wnt3a ligand, a typical ligand for the canonical Wnt signaling pathway, the MSCs exhibit enhanced proliferation, but decreased apoptosis [82]. However, treatment with secreted frizzled-related protein 3, a canonical Wnt signaling inhibitor, had opposite effects on MSCs. Another research group used lithium as a proliferation activator [83] to target glycogen synthase kinase-3*β* (GSK-3*β*), a downstream protein in the canonical Wnt signaling pathway. When BM-derived MSCs were treated with lithium, the proportion of cells in S phase and expression levels of cyclin D1 were significantly increased. In contrast, Wnt5a, a noncanonical Wnt member, has been reported to promote MSC differentiation [84, 85], suggesting that a distinct mechanism of Wnt ligand-mediated MSC signaling might be involved.

Estrogen is the primary female sex hormone and controls the female reproductive system and secondary sex characteristics, indicating its multifunctional roles in many tissues. Similar to many other types of cells, MSCs have estrogen receptor (*α* and *β*), suggesting that estrogen might affect MSC function. Estradiol (E2), a major form of estrogen, was found to affect MSC proliferation [86]. 17-*β* estradiol-pretreated MSCs exhibited a significantly increased proliferation rate in vitro, although it did not alter the MSC phenotypes, including the surface marker expression of MSCs such as CD105 and CD166. Based on these data, 17-*β* estradiol might be a priming agent for enhancing MSC function in practical applications.

### 4.3. Enhancing MSC Homing for Cardiovascular Regeneration

The process of delivering cells to injured tissues is called “homing,” which is induced in response to a diverse array of molecules including chemokines and growth factors. To overcome the insufficient number of homed MSCs, many researchers have investigated the pivotal regulators of MSC homing to ischemic sites.

MSCs strongly express CXC chemokine receptor 4 (CXCR4) on their surface. Stromal-derived factor-1 (SDF-1), which is secreted from ischemic tissues, binds to CXCR4 on the MSC surface, inducing MSC migration to injured sites. In line with this mechanism, MSCs were retrovirally transduced with a CXCR4 overexpression vector and preclinically applied to an MI disease model [87]. As a result, there were an increased cell number of transplanted cells, and echocardiographic imaging of the MI area showed less anterior wall thinning and improvement in the left ventricular (LV) chamber dimensions.

Arachidonic acid (AA) is a polyunsaturated omega-6 fatty acid found in the cell's membrane, and it plays a critical role as a lipid second messenger. Although it has been reported that injured tissues release large amounts of AA, the detailed and precise mechanism is not fully understood. The effect of AA on skin wound healing was studied with human umbilical cord blood-derived MSCs (hUCB-MSCs) [88], and preconditiong hUCB-MSCs with AA improved wound healing, reepithelization, and angiogenesis. AA activates mammalian target of rapamycin complex 2 (mTORC2) and Akt via the GPR40/phosphoinositide 3-kinase (PI3K) signaling pathway. p38 is phosphorylated by PKC*ζ* and it activates Sp1. As a result, membrane type 3-matrix metalloproteinase (MT3-MMP) is stimulated, and eventually the secreted MT3-MMPs degrade fibronectin and facilitate hUCB-MSC migration, suggesting that skin wound healing might be promoted by priming MSCs with AA via MT3-MMP-dependent fibronectin degradation.

An effective cell therapy for MI requires accurate MSC delivery. To enhance MSC homing activity, phage display approaches have been used to screen MI-specific peptide sequences [89]. In a mouse MI model, four peptide sequences were identified, CRPPR, CRKDKC, KSTRKS, and CARSKNKDC, which are synthesized as palmitated derivatives. MI homing peptides-coated MSCs were injected into a mouse model of MI, and the number of migrated MSCs was greater in the coated groups than in the noncoated groups, indicating that the coating of MSCs with homing peptides is a promising therapeutic methodology for treating cardiovascular diseases including MI.

## 5. Priming Strategies for Therapeutic MSCs for Cardiovascular Regeneration

In general, the low survival rate of transplanted stem cells in ischemic myocardium has limited their therapeutic efficacy against ischemic cardiovascular diseases. Accumulating evidence indicates that the pharmacological pretreatment of MSCs ex vivo is a rational approach to reinforce the cells so that they can withstand the ischemic and reperfusion injury environment [90–92], although researchers first should define and clarify potential priming molecules and agents for cardiovascular regeneration.

Curcumin effectively protects MSCs from oxidative stress via regulation of PTEN/Akt/p53 and HO-1 signaling proteins and thereby promotes VEGF release from MSCs, facilitating the enhancement of cardiac function, improving cells retention, and reducing fibrosis in MI hearts [90]. As a conventional inductor, 5-azacytidine (5-AZA) has been used to induce MSC differentiation into cardiomyocytes [53, 93, 94]. Similarly, BMP-2 present in the embryonic heart was used to differentiate ESCs or induce pluripotent stem cells (iPS) into cardiomyocyte-like cells [95, 96], which opens up new possibilities for cardiomyocyte differentiation from MSCs [97].

Angiotensin-II (Ang-II) is peptide hormone that is produced from angiotensin-I (Ang-I) through modifications by angiotensin-converting enzyme (ACE). It is well-known that Ang-II increases blood volume and pressure. MSCs pretreated with Ang-II trigger VEGF production. Based on the effect of Ang-II on VEGF production, the therapeutic efficacy of Ang-II-preconditioned MSCs was investigated [98]. MSCs, isolated from Sprague-Dawley rats, were pretreated with 100 nM of Ang-II for 24 hours and injected into the border zone of the ischemic heart. After 30 days, the restoration efficacy of MSCs was evaluated by measuring angiogenesis, cardiac function, cell differentiation fibrosis, infarct size, and VEGF expression. Ang-II-MSCs exhibited better cardiac function, higher expression of VEGF and von Willebrand factor (vWF), less cardiac fibrosis, and a smaller infarct size. In addition, preconditioning MSCs with Ang-II resulted in an increased survival rate and enhanced tube formation via the upregulation of connexin-43 (Cx43), suggesting that priming MSCs with Ang-II improved their therapeutic efficacy by enhancing angiogenesis and gap junction formation, in addition to the paracrine effect of VEGF against MI.

Fucoidan, a natural compound found in brown algae and seaweed, has various functional properties in biological processes. This sulfated polysaccharide reacts with cytokines and contributes to improving cell functional activity, including antioxidant effects, proliferation, and differentiation. The effects of fucoidan on preconditioned adipose tissue-derived MSCs (ADSCs) were evaluated in a chronic kidney disease (CKD) model [99]. Fucoidan-ADSCs exhibited increased proliferation potential with a significant increase in cell cycle-associated proteins such as cyclin E, cyclin D1, cyclin dependent kinase 2 (CDK 2), and CDK4. When applied to a CKD disease model, the in vivo transplantation of fucoidan-ADSCs enhanced the proliferation, incorporation, and endothelial differentiation of transplanted MSCs in ischemic sites, revealing a novel therapeutic strategy of using MSCs for cardiovascular regeneration.

## 6. Tissue Engineering Strategies: A Powerful Application of MSC-Mediated Vascular Regeneration

Stem cells derived from healthy tissue can be used for tissue regeneration. In studies of cardiac tissue regeneration, researchers originally focused on organ transplantation. However, with the development of stem cell-based technics, stem cells are predicted to be useful for cardiac tissue regeneration. Particularly, MSCs are promising candidates for heart failure treatment because of their unique characteristics. Over the past decade, MSCs have gained attention as a therapeutic approach for treating MI compared with other cell types considered for cardiomyoplasty. MSCs have unique properties that may translate into convenient and extremely effective cell therapy [8, 100]. Recent reports have questioned their “transdifferentiation” potential after injection into the myocardium and suggested the benefits of MSC mechanisms [101]. However, the tissues regenerated by this tissue engineering and widely applied to patients are still very limited, including skin, bone, cartilage, capillary, and periodontal tissues. What are the reasons for such slow advances in clinical applications of tissue engineering? This article gives the brief overview on the current tissue engineering, covering the fundamentals and applications.

### 6.1. Biomaterial-Based Tissue Engineering for Cardiovascular Regeneration

Biomaterials are biofriendly materials that have been engineered to interact with biological systems, and they are capable of protecting transplanted cells against harsh ischemic environments, including low oxygen, nutrient depletion, and severe attack by inflammatory cells [102].

Scaffolds containing silicon dioxide for tissue engineering enhance MSC growth through ERK1/2 activation [103]. Another study showed that the proliferation of hMSCs cultured in media containing 2 or 4 *μ*M silicon was significantly higher than that in control medium, suggesting the enhanced mechanical strength of the medium with silicon may have contribute to this result [104]. Other scaffolds, such as collagen-HA, also enhance MSC attachment and proliferation [105], and collagen I scaffolds exhibit excellent cellular compatibility [106]. Similarly, nanoparticle-containing liposomes were cultured with MSCs to make sheet-like structures [107]. The MSC sheets were layered on the ischemic tissues, and the recovery efficiency was evaluated. The organized structure of MSC sheets provided protection against ischemic limb diseases due to increased blood flow and recovery of angiogenesis, suggesting that tissue engineering scaffolds containing nanoparticles might improve MSC growth and that single-component silica-derived nanoparticles could be advantageous for scaffolds used in stem cell therapy [103, 108]. In addition, various biomaterials have been employed in 3D scaffolds for cultured MSCs, such as chitosan, silk, and alginate [109–111].

Cell-based gene therapy combined with biomaterial-based tissue engineering offers an alternative strategy for therapeutic angiogenesis. Chinese hamster ovary (CHO) cells were transfected with pCDNA3-VEGF-hemagglutinin (HA) vector [112]. The genetically modified CHO cells secreted VEGF and they were enveloped into semipermeable microcapsules. When the microencapsulated VEGF-CHO cells were transplanted into the MI region of rats, the capillary density of the microencapsulated VEGF-CHO cell group was significantly higher than that of the control group, with functional improvement of the injured heart. Wang et al. also examined the effect of the transplantation of microencapsulated Schwann cells with MSCs on angiogenesis [113]. In the harsh microenvironment of the nervous system, Schwann cells secrete VEGF, which enhances neuronal survival [114]. When semipermeable alginate-poly-L-lysine-alginate microcapsules containing Schwann cells and MSCs were applied to an acute myocardial infarction (AMI) rat model, they improved cardiac function because the recipient cells avoided the immune reaction due to the microencapsulation, and they secreted VEGF through small pores on their surface. Another research group investigated genetically modified MSCs that secreted glucagon-like peptide 1 (GLP-1) [115], which regulates blood glucose homeostasis and has a cardioprotective effect in heart disease. When Bead-GLP-1 MSCs were delivered to coronary artery branches in a porcine MI model, the echocardiography results showed improved left ventricular (LV) function, whereas histological analysis showed reduced inflammation and a lower apoptosis rate, indicating that combining the therapeutic strategies of utilizing recombinant GLP-1 and the inherent paracrine stem cell factors of MSCs might be beneficial for clinical application against MI.

**Figure 1:**
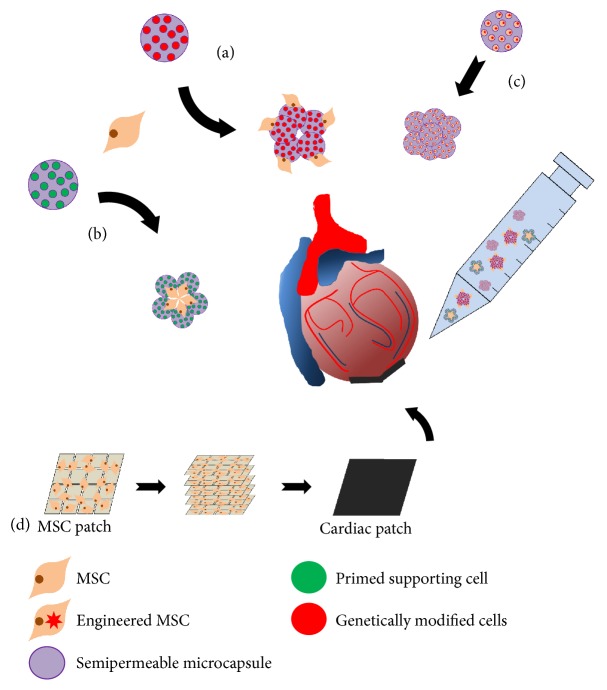
Therapeutic strategies of MSC-based tissue engineering. (a) Genetically modified MSCs are encapsulated into semipermeable microcapsules. This modified MSC secrete cytokine for homed stem cell. (b) Semipermeable microcapsules containing primed supporting cells. Secreted cytokines from primed supporting cells enhance MSC cellular function. (c) Engineered MSCs encapsulated into semipermeable microcapsules. MSCs secreted recombinant hormone such as glucagon-like peptide 1. Each semipermeable microcapsule is transplanted into infracted region of heart. (d) MSCs seeded on biomaterial-based patch. Multilayered cells as a cardiac patch for myocardial infraction.

Recently, cardiac patch-based therapeutics have been suggested as novel noninvasive methods functional strategies for cardiac regeneration because they can overcome the negative aspects of invasive surgery including complications resulting from the altered mechanics of the infarcted heart [116]. Bioengineered cardiac patches are made from a stem cell seeded multilayered scaffold. Briefly, stem cells, including MSCs, cardiac progenitor cells (CPCs), and endothelial progenitor cells (EPCs), grow on the cell-sheet culture system and generate a cell-sheet structure that can be cultured multiple times. Recently, MSC patches were applied to infarcted hearts with a previously described protocol [117]. The engineered MSC sheets represent a tightly adhered meshwork with adhesive agents, including fibronectin and laminin. MSC patches were attached to the ligated left coronary artery (LCA) in rats. Twelve weeks after patch implantation, echocardiography and heart catheterization were performed. The MSC patch implanted group exhibited improved heart function. In addition, neomuscle fibers and neomicrovessels were observed in the ligated LCA, as well as increased levels of angiogenic cytokines (bFGF, vWF, and PDGF-B) and cardioprotective factors (IGF-1 and HGF) in the MSC patch group, indicating the effectiveness of the bioengineered MSC patch as a therapeutic strategy against cardiovascular diseases including MI. (Figure 1). Taken together, studies of potential bioengineered cardiac patches-based therapeutics may improve the therapeutic efficacy of transplanted MSCs to overcome the limited number of multilayer of cardiac patches for cell survival and complications resulting from the altered mechanics of the infarcted heart and improve cell infiltration and migration of transplanted patch-derived MSCs.

### 6.2. Emerging 3D Printing-Based Tissue Engineering for Cardiovascular Regeneration

3D printing is a novel manufacturing technique in which 3D objects can be synthesized. Objects that researchers want to make are captured with computed tomography (CT) or magnetic resonance imaging (MRI). Then, a 3D computer assisted design (CAD) model is developed from the captured objects. Digitally sliced images are generated by a visualized motion program. Finally, the captured objects are printed with a 3D printer, and they can be reprinted as needed [118]. Similar to commercial personal printers, 3D printers require ink. 3D printers, however, can use many types of ink. The human body is composed of various types of tissue. These tissue structures cover a wide range of sizes and stiffness. To meet these different demands, many types of ink have been developed. To develop functional 3D-engineered tissue constructs, various key components should be evaluated.

**Figure 2:**
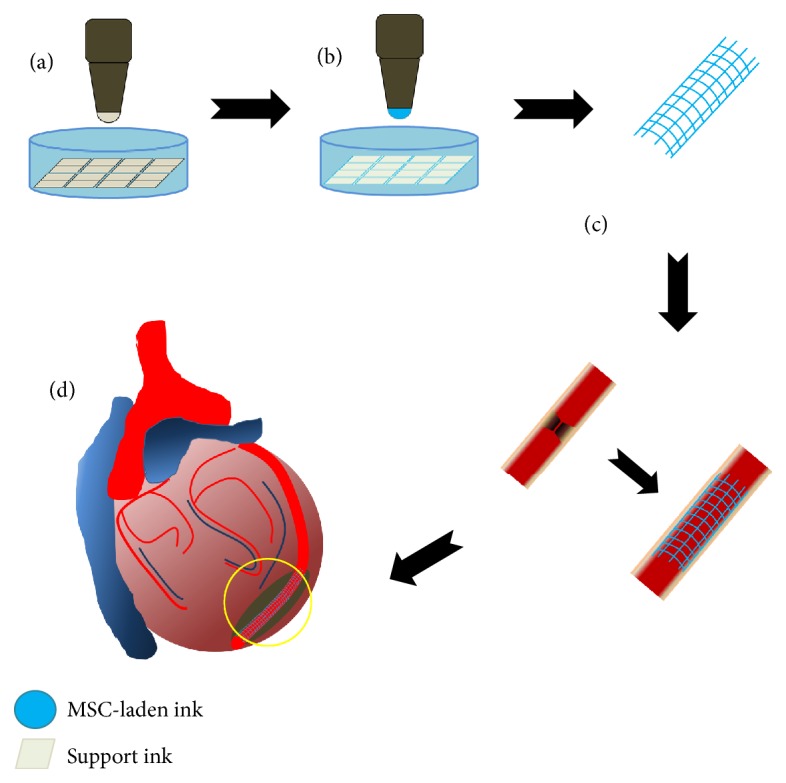
3D printing-based tissue engineering for myocardiac infarction. (a) Casting a support frame with support ink. (b) MSC-laden ink is cast over the support ink. (c)-(d) 3D printing-based engineered tissues could be used for myocardiac infarction treatment.

3D-engineered tissue constructs require several key components, such as cells, extracellular matrix (ECM), and vasculature. Each component supports a biomimetic function of the engineered tissue constructs. Thick vascularized tissues were made by bioprinting with 3D cell-laden ink [119] using two types of ink. Fugitive (vascular) ink contains pluronic and thrombin, and cell-laden ink contains gelatin, fibrinogen, thrombin, transglutaminase, and cells. Fugitive ink is printed on a 3D perfusion chip. Then, cell-laden ink is cast over the printed inks. Gelatin and fibrins are cross-linked by diffused transglutaminase from the molten casting matrix. Upon cooling, the fugitive ink liquefies and is evacuated, leaving behind a pervasive vascular network. hMSCs and human neonatal dermal fibroblasts (hNDFs) are subsequently lined with human umbilical vein endothelial cells (HUVECs). These thick vascularized tissues are actively perfused with growth factors necessary for differentiation of hMSCs. Taken together, 3D printing has gained a considerable amount of attention owing to its ability to provide precise control of the initial structure of tissue-engineered constructs [102], indicating that 3D scaffold architecture and geometric cues play a major role in dictating cell behavior and tissue regeneration (Figure 2).

## 7. Conclusion

MSCs can be readily isolated from various sources in the human body. In addition, MSCs are able to self-replicate for many passages and differentiate into multiple cell lineages, such as osteoblasts, myoblasts, and fibroblasts. Thus, MSCs have become the most practical and prominent therapeutic stem cell. Recently, a number of research groups have focused on applying MSC-based therapies clinically relevant disease models. Based on MSC signaling pathways, natural compounds or chemical drugs are used to improve of MSC functions. Moreover, scientists are working to develop novel materials that are biologically inert. With the advances in technology, it is possible to modulate the microstructure of biomaterials to enable their practical use in medicine. An artificial structure composed of modified biomaterials can enhance MSC proliferation, survival, and differentiation. Tissue engineering technologies such as cell-scaffolds, cell-protein-scaffold architectures made of biomaterials including ECM or hydrogel, and cell patch- and 3D printing-based tissue techniques allow researchers to make artificial versions of human tissues and organs. Because of its numerous applications, a combined therapeutic strategy that includes cell priming and tissue engineering technology is a promising therapeutic approach for cardiovascular regeneration.

## Figures and Tables

**Table 1:** Clinical trials with mesenchymal stem cells (https://clinicaltrials.gov/).

	Study	Year (country)	Study status	Age	Number treated	Phase	Study ID
1	Mesenchymal Stem Cells and Myocardial Ischemia	2010–2014 (France)	Completed	18 years and older	10	Phase 1 Phase 2	NCT01076920

2	Administration of Mesenchymal Stem Cells in Patients with Chronic Ischemic Cardiomyopathy (MESAMI2)	2015-2016 (France)	Ongoing	18 years to 75 years	90	Phase 2	NCT02462330

3	Stem Cell Therapy for Vasculogenesis in Patients with Severe Myocardial Ischemia	2009–2013 (Denmark)	Completed	30 years to 80 years	31	Phase 1 Phase 2	NCT00260338

4	Human Umbilical-Cord-Derived Mesenchymal Stem Cell Therapy in Ischemic Cardiomyopathy	2015–2018 (China)	Ongoing	18 years to 80 years	40	Phase 1 Phase 2	NCT02439541

5	MesenchYmal STROMAL CELL Therapy in Patients with Chronic Myocardial Ischemia (MyStromalCell Trial)	2010–2014 (Denmark)	Completed	30 years to 80 years	60	Phase 2	NCT01449032

6	Safety and Exploratory Efficacy Study of UCMSCs in Patients With Ischemic Heart Disease (SEESUPIHD)	2016-2017 (China)	Ongoing	18 years to 70 years	64	Phase 1 Phase 2	NCT02666391

7	Intracoronary Autologous Mesenchymal Stem Cells Implantation in Patients with Ischemic Dilated Cardiomyopathy	2012–2015 (Malaysia)	Ongoing	35 years to 75 years	80	Phase 2	NCT01720888

8	Therapy of Preconditioned Autologous BMMSCs for Patients With Ischemic Heart Disease	2015–2017 (China)	Ongoing	up to 75 years	200	Phase 1 Phase 2	NCT02504437

9	The TRansendocardial Stem Cell Injection Delivery Effects on Neomyogenesis STudy (The TRIDENT Study)	2013–2017 (USA)	Ongoing	21 years to 90 years	30	Phase 2	NCT02013674

10	Mesenchymal Stem Cell Administration in the Treatment of Coronary Graft Disease in Heart Transplant Patients	2014–2017 (France)	Ongoing	18 years to 80 years	14	Phase 1 Phase 2	NCT02472002

11	Safety and Efficacy of Intracoronary Adult Human Mesenchymal Stem Cells after Acute Myocardial Infarction	2007–2011 (Korea)	Completed	18 years to 70 years	80	Phase 2 Phase 3	NCT01392105

12	Human Umbilical Cord Stroma MSC in Myocardial Infarction	2014–2017 (Turky)	Ongoing	30 years to 80 years	79	Phase 1 Phase 2	NCT02323477

13	Stem Cell Injection to Treat Heart Damage during Open Heart Surgery	2012–2020 (USA)	Ongoing	18 years to 85 years	60	Phase 1	NCT01557543

14	Safety Study of Adult Mesenchymal Stem Cells (MSC) to Treat Acute Myocardial Infarction	2005–2014 (Australia)	Completed	21 years to 85 years	53	Phase 1	NCT00114452

15	RELIEF (A Randomized, Open labEled, muLticenter Trial for Safety and Efficacy of Intracoronary Adult Human Mesenchymal stEm Cells Acute Myocardial inFarction)	2012–2016 (Korea)	Ongoing	20 years to 70 years	135	Phase 3	NCT01652209

16	Intracoronary Human Wharton's Jelly-Derived Mesenchymal Stem Cells (WJ-MSCs) Transfer in Patients with Acute Myocardial Infarction (AMI)	2011–2015 (China)	Completed	18 years and older	160	Phase 2	NCT01291329

17	Ex Vivo Cultured Bone Marrow Derived Allogenic MSCs in AMI	2009–2013 (India)	Completed	20 years to 70 years	20	Phase 1 Phase 2	NCT00883727

18	“ESTIMATION Study” for Endocardial Mesenchymal Stem Cells Implantation in Patients after Acute Myocardial Infarction	2011–2016 (Russia)	Ongoing	30 years to 75 years	50	Phase 3	NCT01394432

19	Prochymal® (Human Adult Stem Cells) Intravenous Infusion following Acute Myocardial Infarction (AMI)	2009–2016 (Australia)	Ongoing	21 years to 85 years	220	Phase 2	NCT00877903

20	Plasmonic Nanophotothermic Therapy of Atherosclerosis	2007–2015 (Russia)	Completed *Has results*	45 years to 65 years	180	Phase 1 Phase 2	NCT01270139

21	The Percutaneous Stem Cell Injection Delivery Effects on Neomyogenesis Pilot Study (The POSEIDON-Pilot Study)	2010–2015 (USA)	Completed *Has results*	21 years to 90 years	31	Phase 1 Phase 2	NCT01087996

22	The Transendocardial Autologous Cells (hMSC or hBMC) in Ischemic Heart Failure Trial (TAC-HFT)	2008–2015 (USA)	Completed *Has results*	21 years to 90 years	65	Phase 1 Phase 2	NCT00768066

23	Safety and Efficacy Study of Stem Cell Transplantation to Treat Dilated Cardiomyopathy	2013–2015 (Slovenia, USA)	Completed	18 years to 80 years	110	Phase 2	NCT00629018

24	Prospective Randomized Study of Mesenchymal Stem Cell Therapy in Patients Undergoing Cardiac Surgery (PROMETHEUS)	2007–2015 (USA)	Completed *Has results*	21 years to 80 years	9	Phase 1 Phase 2	NCT00587990

25	Human Umbilical Cord-derived Mesenchymal Stem Cells with Injectable Collagen Scaffold Transplantation for Chronic Ischemic Cardiomyopathy	2015–2018 (China)	Ongoing	35 years to 65 years	45	Phase 1 Phase 2	NCT02635464

26	The Effect of Mobilized Stem Cell by G-CSF and VEGF Gene Therapy in Patients with Stable Severe Angina Pectoris	2003–2011 (Denmark)	Completed	20 years to 80 years	48	Phase 1 Phase 2	NCT00135850

27	Plasmonic Photothermal and Stem Cell Therapy of Atherosclerosis versus Stenting	2010–2015 (Russia)	Terminated	45 years to 65 years	62	Phase 1	NCT01436123

28	Clinical Trial of Autologous Adipose Tissue Derived Stromal Cell Therapy for Ischemic Heart Failure	2012–2014 (Japan)	Enrolling by invitation	20 years and older	6	—	NCT01709279

29	Safety Study of Allogeneic Mesenchymal Precursor Cell Infusion in MyoCardial Infarction	2012–2015 (USA)	Ongoing	18 years and older	225	Phase 2	NCT01781390

30	PercutaneOus StEm Cell Injection Delivery Effects On Neomyogenesis in Dilated CardioMyopathy (The POSEIDON-DCM Study)	2011–2016 (USA)	Ongoing	18 years to 95 years	36	Phase 1 Phase 2	NCT01392625
